# A Comparative Study on Outcomes of Partial Meniscectomy for Horizontal Cleavage Tear of Medial Meniscus: Complete versus Partial Resection of Inferior Leaf

**DOI:** 10.3390/jcm12041439

**Published:** 2023-02-10

**Authors:** Sang Woo Jeon, Chong Hyuk Choi, Sung-Hwan Kim, Sung-Jae Kim, Kyutae Kang, Min Jung

**Affiliations:** 1 Department of Orthopaedic Surgery, Ewha Womans University Seoul Hospital, Seoul 07804, Republic of Korea; 2 Arthroscopy and Joint Research Institute, Department of Orthopaedic Surgery, Yonsei University College of Medicine, Seoul 03722, Republic of Korea; 3 Department of Orthopaedic Surgery, Yonsei University College of Medicine, Seoul 03722, Republic of Korea

**Keywords:** medial meniscus, horizontal tear, meniscectomy, clinical outcome

## Abstract

The extent to which resection of unstable leaf should be performed in horizontal cleavage meniscus tear has not yet been elucidated. The purpose of this study was to compare the clinical outcomes of partial meniscectomy for horizontal cleavage tear of medial meniscus between complete resection of inferior leaf including the periphery up to the joint capsule and partial resection leaving stable peripheral torn meniscal tissue. A total of 126 patients who underwent partial meniscectomy for horizontal cleavage tear of medial meniscus were divided into two groups: group C (*n* = 34), treated with the complete resection of the inferior leaf; and group P (*n* = 92), treated with partial resection of the inferior leaf. The minimum follow-up duration was 3 years. Functional outcomes were evaluated using the Lysholm knee scoring scale, International Knee Documentation Committee (IKDC) subjective knee evaluation form, and knee injury and osteoarthritis outcome score (KOOS). Radiologic assessments were performed using the IKDC radiographic assessment scale and measurement of the height of the joint space in the medial compartment of the tibiofemoral joint. The functional outcomes including the Lysholm knee, IKDC subjective score, activities of daily living and sport and recreation subscale of KOOS were worse in group C than in group P (*p* < 0.001). The radiologic outcomes including postoperative IKDC radiographic scale (*p* = 0.003) and the postoperative joint space on the affected side (*p* < 0.001) were also worse in group C than in group P. In the horizontal cleavage tear of medial meniscus, complete resection of the inferior leaf including the periphery up to the joint capsule showed inferior clinical outcomes compared with partial resection leaving stable peripheral rim of torn meniscus at minimum 3-year follow-up. If the peripheral part of the inferior leaf is stable in horizontal cleavage tear of medial meniscus, partial resection of the inferior leaf preserving peripheral rim can be recommended.

## 1. Introduction

The meniscus tear, one of the common knee injuries [1], was managed with indiscriminate resection in the early period [1,2]. However, excessive resection of meniscal tissue has been found to predispose the knee joint to arthritis and to result in poor long-term outcomes in patients [3]. Given the crucial role of the meniscus in the knee joint [4], the treatment of meniscal lesions has been increasingly focused on preserving the meniscal tissue [5]. Accordingly, meniscus repair rather than meniscectomy should be the preferred treatment option for meniscus tear [5,6]; however, meniscus repair does not always guarantee good clinical outcomes in every meniscus tear. Meniscectomy can be inevitably performed as the treatment of choice depending on factors affecting the repairability, such as age, chronicity, tissue quality and meniscus vasculature [7]. The principle of meniscectomy is based on minimal resection of the unstable torn meniscal tissue and maximal preservation of the stable remaining meniscus [8,9]. The meniscal width, including the peripheral rim and thickness, should be preserved whenever possible to maintain biomechanical function of the meniscus and protect knee articular cartilage from osteoarthritis progression.

Surgical treatment of the horizontal cleavage tear of the meniscus is also performed according to this principle of minimal resection. When a meniscus has a horizontal cleavage tear, the meniscal tissue is separated into the upper and lower leaves [10]. A horizontal cleavage tear is a type of chronic degenerative meniscal tear that is not considered repairable. Accordingly, a symptomatic horizontal cleavage tear is mostly treated with meniscectomy [11,12]. Resection of a horizontal cleavage tear is usually performed by excising the unstable torn leaf of the upper or lower leaves to leave as much meniscal tissue as possible [12]. However, the extent to which unstable leaves should be excised has not yet been established. If a horizontal cleavage tear progresses to the periphery, resection can be performed completely up to the joint capsule. However, a horizontal cleavage tear at the periphery is often stable, so resection of the horizontal cleavage tear to the periphery may not be necessary. Complete removal of a torn leaf, including peripheral meniscal tissue up to the capsule, can reduce the risk of re-tear, but deteriorated meniscal function and subsequent progression of arthritic change can result from loss of more meniscal tissue and decreased thickness of the remaining meniscus [10,12]. On the other hand, if meniscectomy is performed leaving a peripheral part of the torn meniscus, there could be a concern that the patient’s symptoms may persist or recur [13]. However, to the best of our knowledge, the extent to which resection should be performed in a horizontal cleavage meniscus tear has not yet been elucidated. Therefore, this study aimed to compare the clinical outcomes of partial meniscectomy for horizontal cleavage tears of the medial meniscus between complete resection of the inferior leaf including the periphery up to the joint capsule and partial resection leaving stable peripheral torn meniscal tissue.

## 2. Materials and Methods

### 2.1. Study Participants

**Figure 1:**
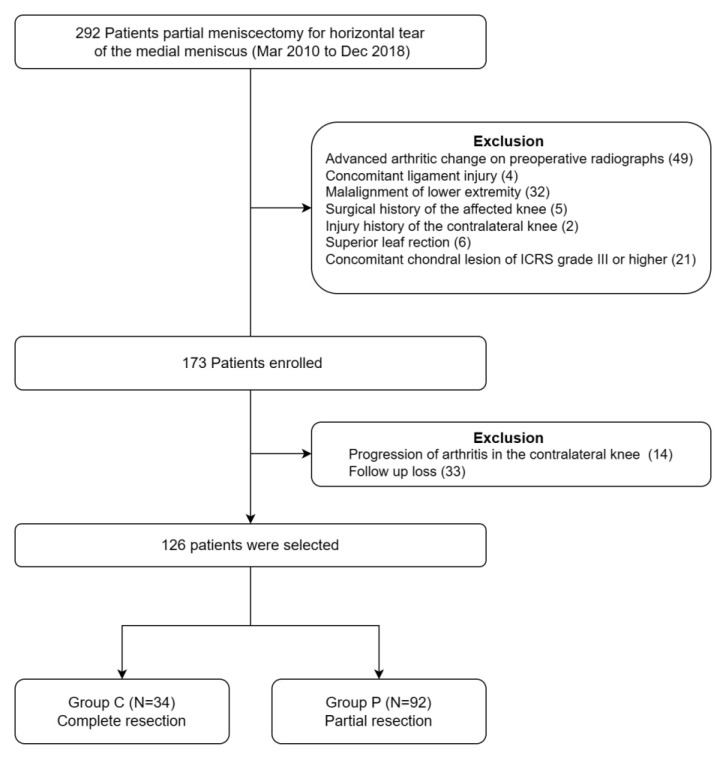
Flowchart of patient inclusion.

A total of 292 patients who underwent partial meniscectomy for horizontal tear of the medial meniscus between March 2010 and December 2018 were retrospectively reviewed after approval from our institutional review board. Patients who met the following criteria were included in the study: (1) medial meniscus tear treated with arthroscopic meniscectomy; (2) case in which the main type of meniscus tear was a horizontal cleavage tear extending to periphery; (3) case in which the unstable inferior leaf was mainly excised out of the superior and inferior leaves of horizontal tear; (4) case in which the superior leaf was left intact or only trimmed; (5) no advanced arthritic change on preoperative radiographs (International Knee Documentation Committee (IKDC) radiologic grade [14] of normal (A) or near normal (B); and (6) minimum follow-up duration of 3 years. The exclusion criteria were as follows: (1) concomitant chondral lesion of International Cartilage Repair Society (ICRS) grade [15] III or higher, confirmed during arthroscopy; (2) concomitant ligament injury; (3) malalignment of lower extremity (normal mechanical axis line passes 8 ± 7 mm medial to the center of the knee joint line on standing hip-knee-ankle radiography [16]; (4) surgical history of the affected knee; (5) injury history of the contralateral knee; and (6) progression of arthritis in the contralateral knee during the postoperative follow-up duration (to exclude the effect of natural degeneration over time). After application of the inclusion and exclusion criteria, 126 patients were included in this study (Figure 1). The statistical power calculated with G*Power version 3.1 (Düsseldorf, Nordrhein-Westfalen, Germany) [17] was 80.6% regarding the postoperative Lysholm knee score.

**Figure 2:**
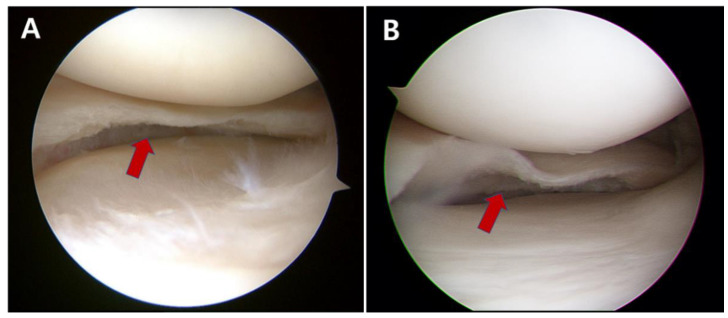
Resection of unstable inferior leaf for horizontal cleavage tear of medial meniscus was performed. (**A**) Complete resection: the inferior leaf of the horizontal cleavage tear was completely excised (red arrow), and the meniscal tissue of the inferior leaf at the torn portion was not left up to the joint capsule. (**B**) Partial resection: the meniscal tissue of the inferior leaf was partially resected, and the stable peripheral area of torn meniscus remained (red arrow).

The included patients were classified into two groups. In the first group, the inferior leaf of the horizontal cleavage tear of the medial meniscus was completely excised, and the meniscal tissue of the inferior leaf at the torn portion did not remain up to the joint capsule (complete resection group: group C) (Figure 2A). In the second group, the meniscal tissue of the inferior leaf was partially resected, and the torn meniscal tissue of the peripheral marginal area remained (partial resection group: group P) (Figure 2B). Each meniscectomy was performed according to the principle of excising only the unstably torn part of the meniscus to preserve as much stable meniscal tissue as possible, while the decision to perform complete or partial resection was determined based on the stability of the torn peripheral area of the meniscus. Complete resection was performed if the unstable part of the torn inferior leaf extended to the periphery in contact with the capsule. Partial resection was performed when the peripheral part of the inferior leaf was confirmed as stable and without degeneration. In performing partial resection, the peripheral part of the inferior leaf was left without complete excision even though the peripheral parts of the superior and inferior leaves were separated. The stability of the torn inferior leaf and whether or not complete resection was necessary were intraoperatively determined with a graduated probe during the arthroscopic procedure [18].

### 2.2. Clinical Assessment

Clinical assessments were conducted at 6 months preoperatively, at 1 year postoperatively, and annually thereafter. Clinical functions were evaluated using the Lysholm knee scoring scale, the IKDC subjective knee evaluation form, and knee injury and osteoarthritis outcome score (KOOS). The Lysholm knee score and IKDC subjective score were classified into the following four grades based on previous studies (Lysholm knee score [19]: excellent = 95–100; good = 84–94; fair = 65–83; and poor <65; IKDC subjective score [20]: excellent = 90–100; good = 80–89; fair = 70–79; and poor <70). The subscales of the KOOS including pain, other symptoms, activities of daily living (ADL), sport and recreation function (Sport/Rec) and knee-related quality of life (QOL) were also evaluated [21].

**Figure 3:**
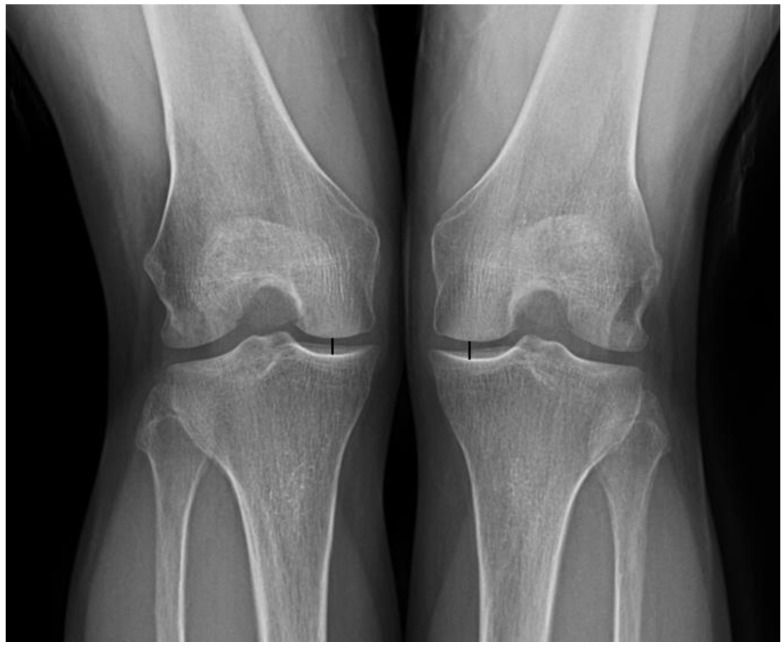
The height of joint space in the medial compartment of the tibiofemoral joint was measured with weight-bearing posteroanterior view taken at 45° of flexion. The distance between the center of the medial femoral condyle and the center of the medial tibial plateau were measured bilaterally (black line).

Radiologic assessment was performed using the IKDC radiographic assessment scale [22,23] and measurement of the height of the joint space in the medial compartment of the tibiofemoral joint. The anteroposterior view, lateral view, and posteroanterior view at 45° of flexion were obtained under weight-bearing conditions along with a Merchant view. The IKDC radiographic assessment scale was classified into four grades as follows: A = normal, no joint space narrowing; B = nearly normal, >4 mm joint space, small osteophytes, slight sclerosis, or femoral condyle flattening; C = abnormal, 2–4 mm joint space; D = severely abnormal, <2 mm joint space. The height of joint space in the medial compartment of the tibiofemoral joint was measured with weight-bearing posteroanterior views taken at 45° of flexion using the methods presented by Shelbourne et al. (Figure 3) [24]. The distance between the center of the medial femoral condyle and the center of the medial tibial plateau were measured bilaterally using a picture archiving and communication system (Centricity PACS, GE Medical System Information Technologies, Milwaukee, WI, USA). The postoperative height was divided by the preoperative height to obtain the rate of joint height loss. Joint heights were measured by two different orthopedic surgeons, and the mean of two measurements was used as the representative value.

### 2.3. Statistical Analysis

The Kolmogorov–Smirnov test [25] was used to evaluate whether the variables followed a normal distribution, and the Levene’s test [26] was used to assess the homogeneity of variance. The two-sample *t*-test or Mann–Whitney U test was used to compare continuous variables between the groups according to the normality test. The paired *t*-test or Wilcoxon signed-rank test was used to compare preoperative and postoperative continuous variables. Chi-square or Fisher’s exact test was used to compare categorical data. Interobserver reliability in the measurement of the height of the joint space in the medial compartment of the tibiofemoral joint was assessed using the intraclass correlation set at a 95% confidence interval. A *p* value of < 0.05 was considered statistically significant. Statistical analyses were performed using SPSS software (version 26.0; IBM Inc, Armonk, New York, USA, 2019).

## 3. Results

The patients’ average age at the time of surgery was 53.4 and 49.1 years in group C and group P, respectively. Group C consisted of 34 patients (21 male, 13 female), while group P consisted of 92 patients (50 male, 42 female). The mean follow-up period was 44.2 and 42.4 months in groups C and P, respectively. There was no statistically significant difference in demographic data and meniscal tear pattern between the groups (*p* > 0.05) (Table 1). Preoperative variables related to the knee joint function, such as Lysholm knee score, IKDC subjective score and five subscales of KOOS, showed no statistically significant difference between the groups (*p* > 0.05) (Table 2). Preoperative radiologic assessment variables including IKDC radiographic scale and height of joint space also did not differ between the groups (*p* > 0.05) (Table 2).

**Figure 4:**
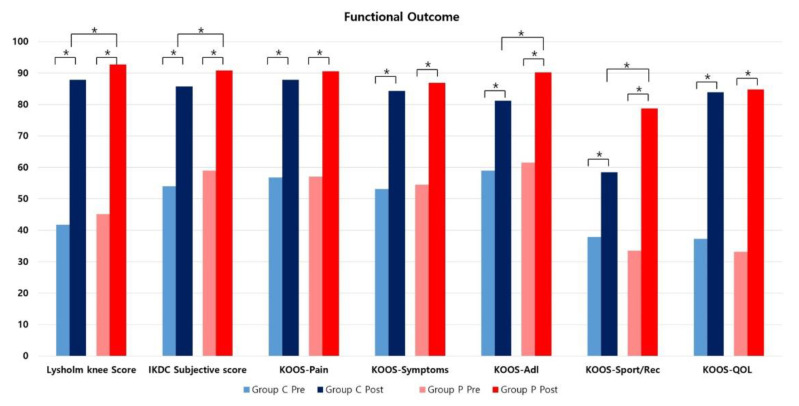
Comparison of functional outcomes for preoperative and postoperative values within groups and postoperative values between the groups. * *p* < 0.05.

**Figure 5:**
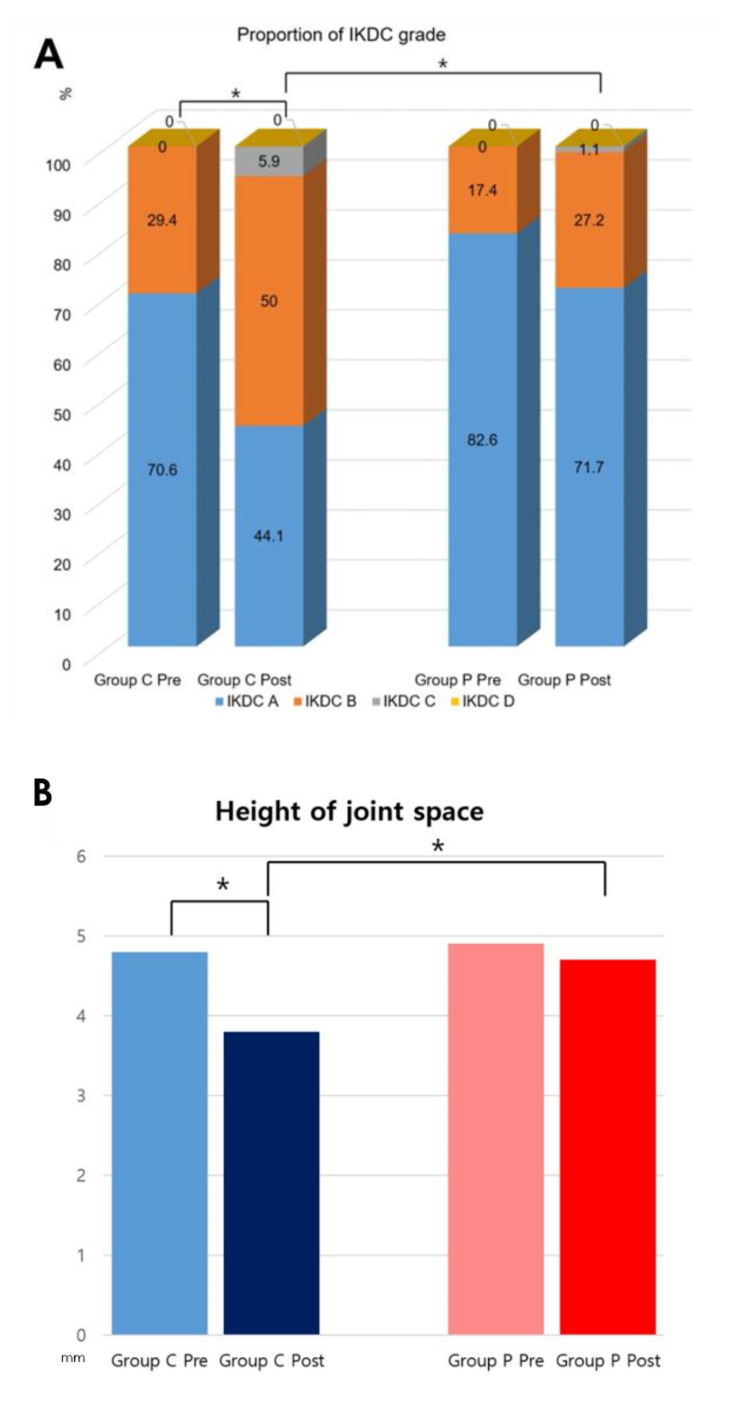
(**A**) Comparison of proportions of IKDC radiographic scale for preoperative and postoperative values within groups and postoperative values between the groups. (**B**) Comparison of the heights of the joint space at the affected side for preoperative and postoperative values within groups and postoperative values between groups. Pre, preoperative; Post, postoperative. * *p* < 0.05.

Comparison between the preoperative and postoperative values of functional outcomes showed significant improvement after meniscectomy in both groups (Table 3, Figure 4). The mean Lysholm knee score improved from 41.7 to 87.8 (*p* = 0.005, effect size 1.25) and from 45.1 to 92.7 (*p* < 0.001, effect size 1.83) after surgery in group C and group P, respectively. The mean IKDC subjective score improved from 54 to 85.7 (*p* < 0.001, effect size 1.49) and from 59 to 90.8 (*p* = 0.014, effect size 1.75) after surgery in group C and group P, respectively. The grade distribution of the Lysholm knee score and IKDC subjective score also improved significantly (*p* < 0.05). All five subscales of KOOS showed significantly improved results in both groups (*p* < 0.05, effect size 0.53 to 1.8). In comparison of radiologic outcomes between the preoperative and postoperative status, the IKDC radiographic scale and height of joint space at the affected side showed significantly worse results only in group C (*p* < 0.001) (Table 4, Figure 5). The radiologic outcomes in group P did not show a statistically significant difference before and after surgery (*p* > 0.05).

In comparison of postoperative functional outcomes between the groups, there was a significant difference in the Lysholm knee score (group C = 87.8 ± 11.2, group P = 92.7 ± 7.4, *p* < 0.001, effect size 0.51) and IKDC subjective score (group C = 85.7 ± 5.9, group P = 90.8 ± 6.7, *p* < 0.001, effect size 0.80). Among the KOOS subscales, only the ADL subscale (group C = 81.2 ± 8.3, group P = 90.2 ± 7.3, *p* < 0.001, effect size 1.15) and Sport/Rec subscale (group C = 58.4 ± 12.1, group P = 78.7 ± 11.7, *p* < 0.001, effect size 1.70) differed significantly between the groups (Table 3, Figure 4). Comparison of postoperative radiologic outcomes between the groups showed a statistically significant difference in the IKDC radiographic scale (A = 15 (44.1%), B = 17 (50%), C = 2 (5.9%), and D = 0 (0.0%) in group C; A = 66 (71.7%), B = 25 (27.2%), C = 1 (1.1%), and D = 0 (0.0%) in group P, *p* = 0.003) and height of joint space at the affected side (group C = 3.8 ± 1 mm, group P = 4.7 ± 0.4 mm, *p* < 0.001, effect size 1.18) (Table 4, Figure 5). There was also a significant difference between the groups in the ratio of postoperative to preoperative height of the joint space at the affected side (group C = 80.3 ± 7.6 %, group P = 96.3 ± 6.7 %, *p* < 0.001, effect size 1.23). The intraclass correlation coefficient for interobserver reliability was 0.889 (95% confidence interval (CI), 0.851~0.928) for the height of the joint space.

## 4. Discussion

In treating horizontal cleavage types of meniscus tears, meniscectomy is usually performed by excising the unstable torn leaf of the two upper or lower leaves to leave as much meniscal tissue as possible [12]. However, the extent to which resection of unstable leaf should be performed remains unknown because excessive resection for meniscal tears may exacerbate changes in arthritis, whereas insufficient resection may result in symptom persistence or recurrence. Therefore, this study aimed to investigate the appropriate extent of meniscal resection by comparing the clinical outcomes between complete resection of the unstable leaf of horizontal tear up to the joint capsule and partial resection of inferior leaf leaving stable peripheral rim of the torn meniscus. The results of the study showed that the functional outcomes of the patients who underwent partial resection were better than those of patients who underwent complete resection. In addition, arthritic change and decreased height of joint space were more pronounced in patients who underwent complete resection than those of patients treated with partial resection.

Both the groups of patients who underwent complete and partial resection of the inferior leaf showed significant postoperative improvement in functional outcomes. The results of the present study showed that resection of the unstable torn leaf of the two upper or lower leaves was an effective treatment method for symptomatic horizontal cleavage tears of medial meniscus. These results are consistent with those of previous studies on the clinical outcomes of operative treatment for horizontal cleavage types of meniscus tears [12,27,28]. In addition to the existing results of previous studies, the present study compared patients treated with partial resection leaving a stable torn portion of the peripheral rim and those treated with complete resection of the unstable leaf of the horizontal tear up to the joint capsule, aiming to determine whether a stable peripheral rim of torn meniscus must be excised when it remains after resection of the unstable inner portion in a horizontal cleavage of medial meniscus tear. According to the results, the partial resection group had more improved functional outcomes compared with the complete resection group at minimum 3-year follow-up. The postoperative meniscal function tends to be proportional to the post-resection amount of remnant meniscal tissue [29,30]. A previous long-term follow-up study showed that good or excellent results were obtained in 88% of the patients at 15 years after arthroscopic partial meniscectomy [31]. Previous in vitro studies also have reported a correlation between the amount of meniscus tissue removed and an immediate increase in joint contact pressure resulting in change of joint pressure distribution and remodeling of the joint [29]. Not only the amount of meniscal tissue remaining, but also the structural state of remnant is important to avoid deterioration of the joint. In particular, preservation of the peripheral meniscal rim is critical for maintenance of meniscal function [30]. Discontinuity of the peripheral rim leads to loss of function of the meniscus as a load distributing structure [32]. Hoop stresses generated by the axial forces across the knee joint compress the meniscus and are converted to tensile stresses along the circumferential collagen fibers of the meniscus [33]. The circumferential fiber of the peripheral meniscal rim plays an important role in maintaining hoop tension [34]. In the partial resection group of this study, the horizontal cleavage tears in the peripheral area were extended to the joint capsule, but since the continuity of the peripheral circumferential fibers was preserved, it is thought that the stable peripheral rim of a horizontal tear contributed to maintenance of the meniscal hoop function [4]. Maintenance of the hoop function could lead to joint preservation and improved functional outcomes. Accordingly, the functional outcomes improved more in the partial resection group, in which only the unstable inner portion was removed, and the stable peripheral rim remained, than in the complete resection group. These results were also in accordance with the meniscectomy principle of minimal resection of the unstable torn portion of the meniscus and preservation of maximum stable meniscal tissue, showing that leaving a stable meniscal tear did not lead to symptom exacerbation.

Another notable finding related to the functional results in the present study was that activities of daily living, sport and recreation function scores among the subscales of KOOS were particularly lower in the complete resection group than in the partial resection group. The activities of daily living subscale in KOOS mainly consists of items that evaluate performance related to knee joint movement and weight-bearing in daily life. This subscale includes knee flexion with weight-bearing on the lower extremities, including climbing stairs or getting up from sitting. The sport and recreation function subscale in KOOS consists of more difficult activities, including weight-bearing squat, kneeling with high flexion of knees causing axial pressure in the joints, running, jumping and pivoting. The activities of daily living and sport and recreation function subscales of KOOS focus on more active behaviors demanding a wide range of knee joint motion with full weight-bearing on lower extremity than other subscales such as symptoms, stiffness, and pain. The results of this study suggest that patients who were treated with partial resection outperformed those treated with complete resection in terms of more active behaviors. It can be thought that maintenance of joint function through preservation of meniscal tissue contributed to the improvement in such active functions.

Regarding radiologic outcomes, comparison between the preoperative and postoperative status showed that only the complete resection group had significantly worse results after surgery on the IKDC radiographic scale and change in the height of joint space at the affected side. Comparison of postoperative status between the groups also showed that the complete resection group had significantly worse outcomes than the partial resection group in terms of the IKDC radiographic scale and height of joint space at the affected side. This suggests that the progression of arthritic change occurred more distinctly in the complete resection group. According to a previous study [12], when the entire single leaflet including the peripheral rim was completely resected up to the joint capsule in the horizontal cleavage type of meniscus tear, only half of the load distribution function of the intact meniscus remained. As a result, the tibiofemoral joint contact area was reduced by 40%, the mean pressure was increased by 24%, and the peak pressure was increased by 10% compared with the intact meniscus. On the other hand, a previous study [35] on the biomechanical effect of meniscectomy noted that when the inferior leaflet was resected to the distal third in the posterior third of the medial meniscus, the peak pressure and contact area did not differ significantly between the native meniscus and inferior leaflet resected meniscus. The present study also showed that there was no significant progression of arthritis or reduction in the height of joint space in the partial resection group, contrary to the progression of arthritis in the complete resection group. It can be thought that maintenance of the hoop function resulting from preservation of the meniscus peripheral rim contributed to prevention of the progression of arthritis [36]. Since the progression of arthritis can lead to a gradual and continuous deterioration in joint function over time, it is necessary to prevent exacerbation of arthritis through partial resection and preservation of the meniscal tissue.

This study had some inherent limitations that warrant review before definite conclusions can be drawn. First, this study was based on retrospective data collection and analysis, which could have introduced patient selection bias. Second, approximately 1/3 fewer patients were included in the complete resection group than in the partial resection group. The reliability of the results could have been influenced by the small sample size. However, the statistical power was calculated to be high (greater than 80%) for comparison of the functional score between the groups, which was the main result of this study. Third, the reason for complete resection of the inferior leaf in group C was that the unstable horizontal cleavage tear had progressed to the peripheral area in contact with the joint capsule. The more extensive unstable tear of the meniscus itself could result in poor postoperative outcomes. Fourth, although significant differences were noted in the postoperative functional outcomes between the groups, they were not substantial. The differences between the groups in the Lysholm knee score, IKDC subjective score and subscales of KOOS were less than the reported minimal clinically important differences (MCIDs). The MCIDs of the Lysholm knee score, IKDC subjective score, and KOOS-ADL were reported to be 8.9, 11.5, and 11.4, respectively, in previous studies [23,37]. A further study including long-term follow-up is needed to achieve more conclusive results.

## 5. Conclusions

In horizontal cleavage tears of the medial meniscus, complete resection of the inferior leaf showed inferior clinical outcomes compared to partial resection at minimum 3-year follow-up. Especially in terms of functional outcome related to activities, the partial resection group showed better outcomes than the complete resection group at minimum 3-year follow-up. The radiologic outcomes related to arthritic change were also less progressed in the partial resection group than in the complete resection group at minimum 3-year follow-up. If the peripheral part of the inferior leaf is stable in a horizontal cleavage tear of the medial meniscus, partial resection of the inferior leaf preserving peripheral rim can be recommended.

## Figures and Tables

**Table 1:** Demographic Data of Patients and the Pattern of Meniscus Tear.

Variables	Group C (*n* = 34)	Group P (*n* = 92)	*p*-Value
Age (years) ^†^	53.4 ± 11.9	49.1 ± 11	0.078
Sex ^‡^			0.458
Male	21 (61.8%)	50 (54.3%)	
Female	13 (38.2%)	42 (45.7%)	
Body mass index (kg/m^2^) ^†^	24.6 ± 3.6	24.8 ± 3.6	0.723
Affected side ^‡^			0.098
Right	23 (67.6%)	47 (51.1%)	
Left	11 (32.4%)	45 (48.9%)	
Follow-up period (months) ^†^	44.2 ± 16	42.4 ± 16	0.573
Type of tear ^‡^			0.380
Only horizontal tear	14 (41.2%)	57 (62%)	
Complex tear including horizontal tear	20 (58.8%)	35 (38%)	
Location of tear ^‡^			0.278
Posterior horn	14(41.2%)	54(58.7%)	
Body	2(5.9%)	3(3.3%)	
Body and posterior horn	18(52.9%)	35(38%)	

^†^ The values are given as mean ± standard deviation. ^‡^ The values are given as *n* (%).

**Table 2:** Comparison of preoperative variables between the groups.

Variables	Group C (*n* = 34)	Group P (*n* = 92)	*p*-Value
Lysholm knee score ^†^	41.7 ± 16.3	45.1 ± 16.7	0.815
Excellent ^‡^	0 (0%)	0 (0%)	0.933
Good ^‡^	2 (5.9%)	6 (6.5%)	
Fair ^‡^	3 (8.8%)	10 (10.9%)	
Poor ^‡^	29 (85.3%)	76 (82.6%)	
IKDC subjective score ^†^	54 ± 12.4	59 ± 11.9	0.138
Excellent ^‡^	0 (0%)	1 (1.1%)	0.495
Good ^‡^	1 (2.9%)	10 (10.9%)	
Fair ^‡^	5 (14.7%)	13 (14.1%)	
Poor ^‡^	28 (82.4%)	68 (73.9%)	
KOOS—Pain ^†^	56.8 ± 19.3	57 ± 18.9	0.868
KOOS—Symptoms ^†^	53.1 ± 19.5	54.5 ± 18.1	0.761
KOOS—ADL ^†^	59 ± 21	61.5 ± 20.5	0.907
KOOS—Sport/Rec ^†^	37.9 ± 24.7	33.5 ± 24.6	0.969
KOOS—QOL ^†^	37.3 ± 23.2	35.2 ± 21.2	0.482
IKDC radiographic scale ^‡^			0.139
A	24(70.6%)	76(82.6%)	
B	10(29.4%)	16(17.4%)	
Height of joint space, mm			
Affected side ^†^	4.8 ± 0.5	4.9 ± 0.4	0.219
Unaffected side ^†^	4.9 ± 0.4	4.9 ± 0.4	0.154

IKDC, International Knee Documentation Committee; KOOS, Knee Injury and Osteoarthritis Outcome Score; ADL, activities of daily living; Sport/Rec, sport and recreation function; QOL, knee-related quality of life. ^†^ The values are given as mean ± standard deviation. ^‡^ The values are given as *n* (%).

**Table 3:** Comparison of functional outcomes between the preoperative and postoperative values in each group.

Variables	Group C (*n* = 34)			Group P (*n* = 92)			
	Preoperative Value	Postoperative Value	*P*-Value ^a^	Preoperative Value	Postoperative Value	*P*-Value ^a^	*P*-Value ^b^
Lysholm knee score ^†^	41.7 ± 16.3	87.8 ± 11.2	0.005	45.1 ± 16.7	92.7 ± 7.4	<0.001	<0.001
Excellent ^‡^	0 (0%)	14 (41.2%)	0.034	0 (0%)	53 (57.6%)	0.023	0.450
Good ^‡^	2 (5.9%)	15 (44.1%)		6 (6.5%)	31 (33.7%)		
Fair ^‡^	3 (8.8%)	4 (11.8%)		10 (10.9%)	8 (8.7%)		
Poor ^‡^	29 (85.3%)	1 (2.9%)		76 (82.6%)	0 (0%)		
IKDC subjective score ^†^	54 ± 12.4	85.7 ± 5.9	<0.001	59 ± 11.9	90.8 ± 6.7	0.014	<0.001
Excellent ^‡^	0 (0%)	16 (47.1%)	<0.001	1 (1.1%)	54 (58.7%)	0.018	0.265
Good ^‡^	1 (2.9%)	15 (44.1%)		10 (10.9%)	34 (37%)		
Fair ^‡^	5 (14.7%)	2 (5.9%)		13 (14.1%)	3 (3.3%)		
Poor ^‡^	28 (82.4%)	1 (2.9%)		68 (73.9%)	1 (1.1%)		
KOOS—Pain ^†^	56.8 ± 19.3	87.8 ± 17.1	<0.001	57 ± 18.9	90.5 ± 16.8	0.010	0.294
KOOS—Symptoms ^†^	53.1 ± 19.5	84.3 ± 7.1	<0.001	54.5 ± 18.1	86.9 ± 6.9	<0.001	0.115
KOOS—ADL ^†^	59 ± 21	81.2 ± 8.3	0.013	61.5 ± 20.5	90.2 ± 7.3	0.017	<0.001
KOOS—Sport/Rec ^†^	37.9 ± 24.7	58.4 ± 12.1	0.024	33.5 ± 24.6	78.7 ± 11.7	0.031	<0.001
KOOS—QOL ^†^	37.3 ± 23.2	83.9 ± 11.8	<0.001	35.2 ± 21.2	84.8 ± 6.4	<0.001	0.665

IKDC, International Knee Documentation Committee; KOOS, Knee Injury and Osteoarthritis Outcome Score; ADL, activities of daily living; Sport/Rec, sport and recreation function; QOL, knee-related quality of life. ^†^ The values are given as mean ± standard deviation. ^‡^ The values are given as *n* (%). ^a^ *P*-value: comparing preoperative and postoperative values. ^b^ *P*-value: comparing postoperative values between groups C and P.

**Table 4:** Comparison of radiologic outcomes between the preoperative and postoperative values in each group.

Variables	Group C (*n* = 34)			Group P (*n* = 92)			
	Preoperative Value	Postoperative Value	*P*-Value ^a^	Preoperative Value	Postoperative Value	*P*-Value ^a^	*P*-Value ^b^
IKDC radiographic scale ^‡^			<0.001			0.152	0.003
A	24(70.6%)	15 (44.1%)		76(82.6%)	66 (71.7%)		
B	10(29.4%)	17 (50%)		16(17.4%)	25 (27.2%)		
C	0(0%)	2 (5.9%)		0(0%)	1 (1.1%)		
D	0(0%)	0 (0%)		0(0%)	0 (0%)		
Height of joint space (mm) ^†^							
Affected side	4.8 ± 0.5	3.8 ± 1	<0.001	4.9 ± 0.4	4.7 ± 0.4	0.184	<0.001
Unaffected side	4.9 ± 0.4	4.6 ± 0.4	0.273	4.9 ± 0.4	4.8 ± 0.6	0.312	0.352

IKDC, International Knee Documentation Committee. ^†^ The values are given as mean ± standard deviation. ^‡^ The values are given as *n* (%). ^a^ *P*-value: comparing preoperative and postoperative values. ^b^ *P*-value: comparing postoperative values between groups C and P.

## Data Availability

The data presented in this study may be available on request from the corresponding author. The data are not publicly available due to privacy and ethical considerations.
